# Opposite Effects of Methanandamide on Lipopolysaccharide-Induced Prostaglandin E2 and F2α Synthesis in Uterine Explants from Pregnant Mice

**DOI:** 10.1371/journal.pone.0039532

**Published:** 2012-07-05

**Authors:** Claudia A. Vercelli, Julieta Aisemberg, Maximiliano Cella, Ana Inés Salazar, Manuel L. Wolfson, Ana M. Franchi

**Affiliations:** Laboratory of Physiopathology of Pregnancy and Labor, Center for Pharmacological and Botanical Studies (National Research Council – School of Medicine, University of Buenos Aires), Ciudad Autónoma de Buenos Aires (CABA), Buenos Aires, Argentina; Konkuk University, Republic of Korea

## Abstract

Prostaglandins (PG) are effective abortifacients and are important mediators of lipopolisaccharide (LPS)-induced embryonic resorption (ER). Besides, anandamide (AEA) has been described as one of the major endocannabinoids present in the uterus suggesting that it might play a role in reproduction. It has been reported that high levels of AEA are associated with pregnancy failure and that LPS increases AEA production. Also, it has been observed that AEA modulates PG production in different tissues. In this sense, we studied whether LPS-induced PG production is modulated by AEA and we also assessed the effect of this endocannabinoid on PG metabolism in an *in vitro* model. Uterine explants from BALB/c implantation sites were cultured in the presence of LPS plus cannabinoid receptor (CB) specific antagonists and PG production was assessed. Then, we studied the effect of exogenous AEA on different steps of PG metabolic pathway. We showed that AEA is involved in LPS-induced PG biosynthesis. Also, we observed that AEA exerts opposite effects on PGE_2_ and PGF_2α_ biosynthesis, by inhibiting PGE_2_ production and increasing PGF_2α_ levels. We suggest that AEA could be involved in the mechanisms implicated in LPS-induced ER. A better understanding of how AEA could be affecting ER could help developing specific interventions to prevent this pathology.

## Introduction

Intrauterine infection plays a major role in the pathogenesis of early pregnancy loss. It has been reported that Gram-negative organisms induce preterm labor and embryonic loss by triggering the release of various proinflammatory molecules, such as cytokines, growth factors and prostaglandins [1], [2], [3]. Prostaglandins are important paracrine regulators of uterine function in normal and pathological pregnancies and are used clinically to induce abortion and stimulate parturition [4]. Our previous results [1] showed that *in vivo* administration of lipopolysaccharide (LPS), a component of Gram-negative bacteria, increased prostaglandin E_2_ (PGE_2_) and prostaglandin F_2α_ (PGF_2α_) production in the uterus of early pregnant mice. We have also observed that, in mice challenge with LPS, the *in vivo* administration of cyclooxygenase (COX) inhibitors prevented LPS-induced embryonic resorption (ER).

Anandamide (arachidonoylethanolamide, AEA) belongs to a group of endogenous lipids termed “endocannabinoids” [5] and is an agonist of type-1 (CB1) and type-2 (CB2) cannabinoid receptors. It has been described as one of the major endocannabinoids present in the uterus and this suggests that it might play a role in reproduction [6]. It has been reported that low levels of AEA are beneficial for implantation and trophoblast outgrowth while increased AEA concentrations are associated with retarded embryo development, fetal loss and pregnancy failure [7]. Previous results indicate that LPS increases AEA levels in human peripheral lymphocytes [8] and in murine macrophages [9]. Our previous results suggest that LPS could be increasing AEA levels in uterine explants by inhibiting its degradation and also by enhancing NAPE-PLD expression, one of its synthesizing enzymes [10]. In addition, recent research has revealed that AEA regulates prostaglandin production in human gestational tissues, cerebral microvascular endothelium and in rat pheochromocytoma PC12 cells [11], [12], [13]. Although several lines of evidence indicate that both, cannabinoids and AEA, stimulate arachidonic acid (AA) release in several tissues [14], [15], [16], the relationship between endocannabinoids and PG metabolism is not fully understood. In the present study we investigated whether LPS-induced PG production is modulated by AEA and we also determined the effect of this endocannabinoid on PG biosynthesis and catabolism in uterine explants from pregnant mice.

## Materials and Methods

### Animals

BALB/c 8- to 12-week-old virgin female mice were paired with 8- to 12-week-old BALB/c males and the day of appearance of a coital plug was taken as day 0 of pregnancy. Animals were housed in cages under controlled conditions of light (14 h light, 10 h dark) and temperature (23–25°C) and received murine chow and water *ad libitum*. Mice were sacrificed by cervical dislocation on day 7 of pregnancy and in each implantation site the uterus and the decidua were separated. Uterine explants (composed of myometrial cells and uterine adventitia) were weighed and culture as described below.

**Figure 1:**
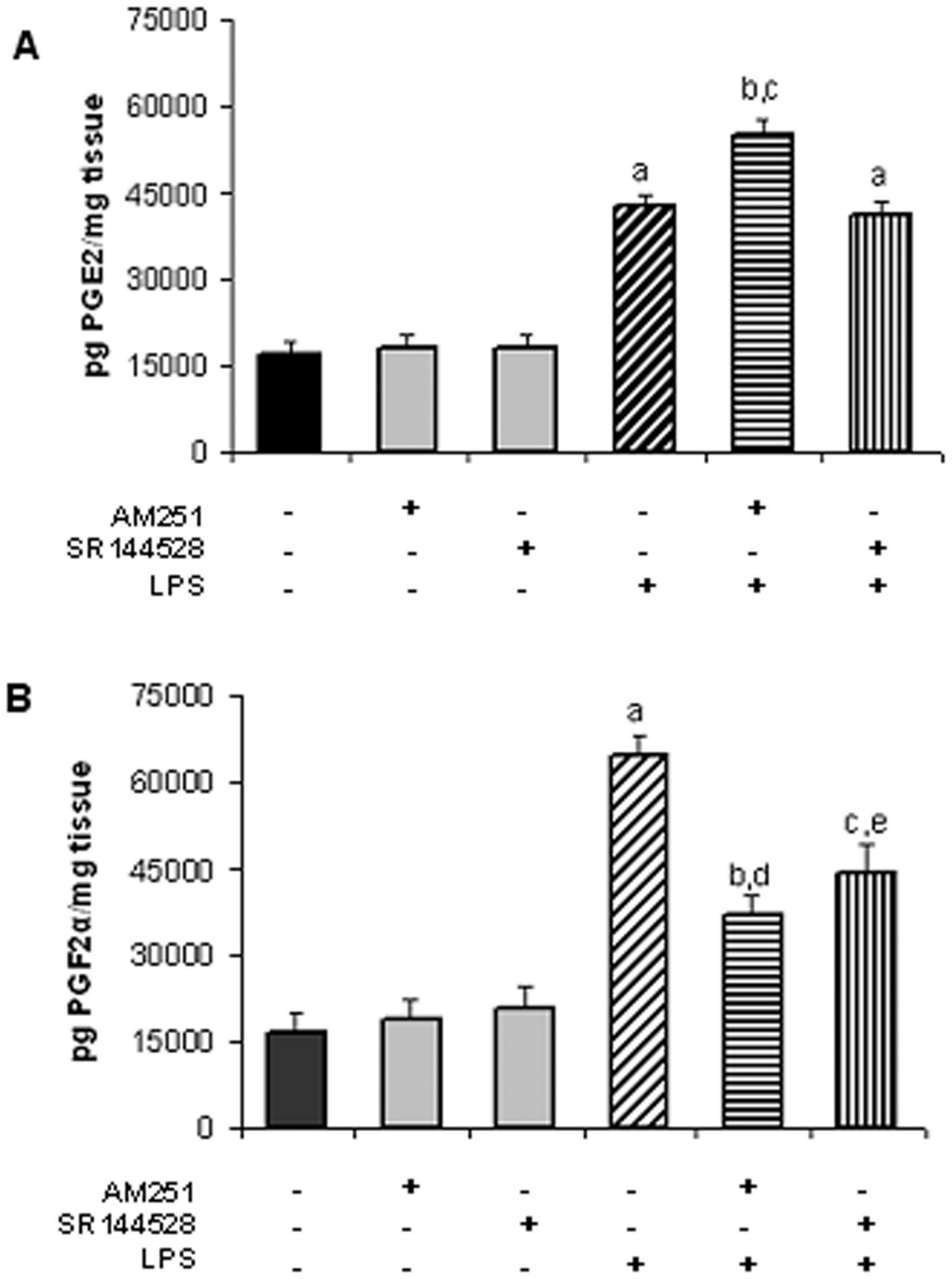
LPS-induced PGE_2_ and PGF_2α_ production is mediated by endocannabinoids. A) PGE_2_ production for uterine explants incubated with LPS (1 ug/ml) and AM251 (10 nM) or SR144528 (10 nM) for 24 h. ANOVA test. a, p<0.001 vs control or AM251 or SR144528; b, p<0.001 vs control or AM251; c, p<0.001 vs LPS. n = 6. B) PGF_2α_ production for uterine explants incubated with LPS (1 ug/ml) and AM251 (10 nM) or SR144528 (100 nM) for 24 h. ANOVA test. a, p<0.001 vs control or AM251 or SR144528; b, p<0.01 vs control or AM251; c, p<0.001 vs control or SR144528; d, p<0.001 vs LPS; e, p<0.01 vs LPS. n = 5.

### Ethics Statement

The experimental procedures reported here were approved by the Animal Care Committee of the Center for Pharmacological and Botanical Studies of the National Research Council (CEFYBO - CONICET) and were carried out in accordance with the Guide for Care and Use of Laboratory Animals (NIH).

### Culture of Uterine Explants from Implantation Sites

We established the optimal culture conditions in a previous work [10]. Briefly, uterine explants were weighed and cultured in wells that contained DMEM supplemented with 10% FCS and 1% antibiotic-antimycotic solution. Tissues were maintained for 24 h in 5% CO_2_ in air at 37°C and then culture supernatants and explants were immediately frozen at –70°C until use.

**Figure 2:**
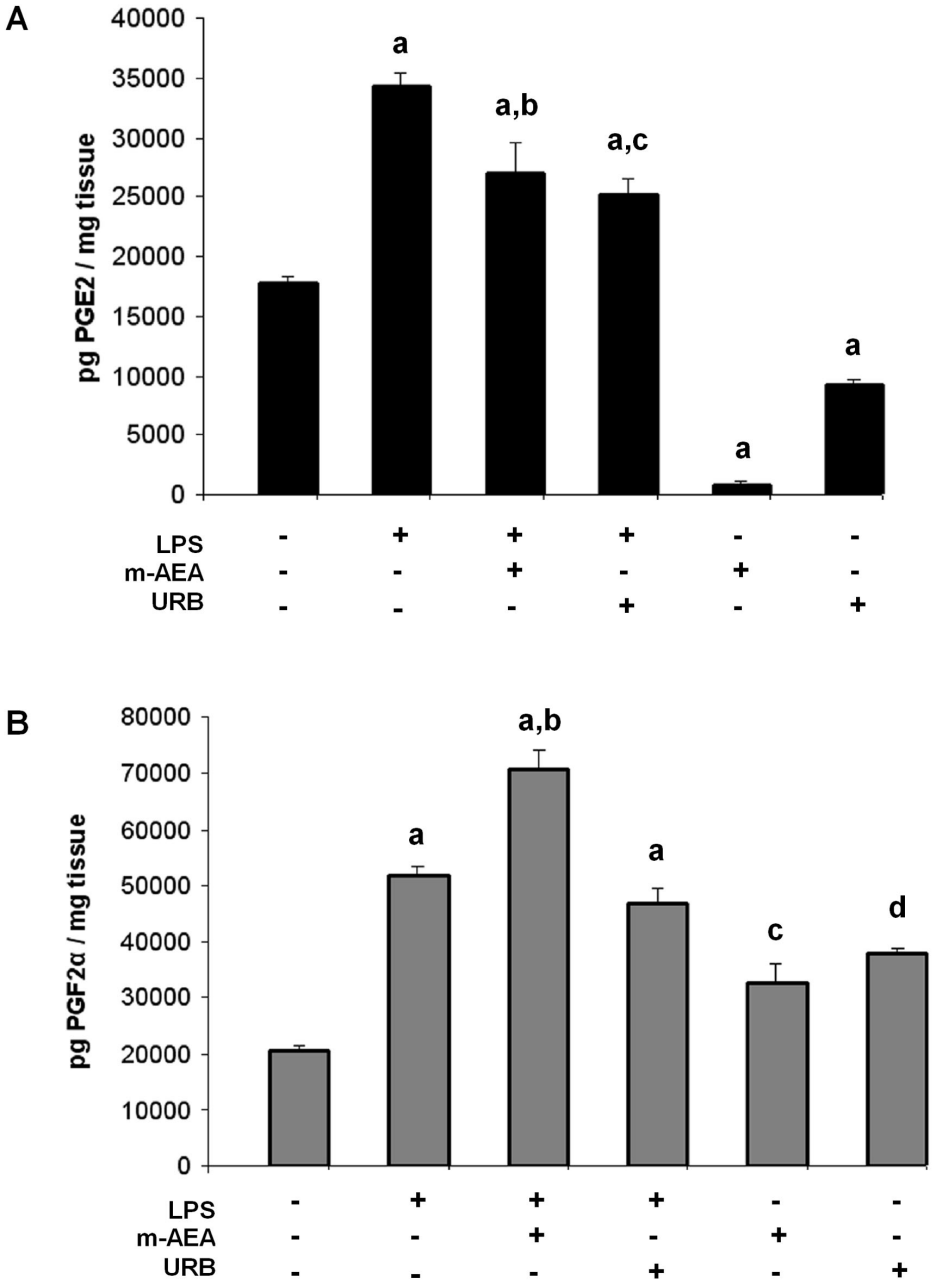
AEA mediates LPS-induced PG production. A) PGE_2_ production for uterine explants incubated with LPS (1 ug/ml), m-AEA (100 nM), URB597 (1 uM), LPS plus m-AEA (100 nM) or LPS plus URB597 (1 uM) for 24 h. ANOVA test. a, p<0.001 vs control; b, p<0.01 vs LPS; c, p<0.001 vs LPS. n = 7. B) PGF_2α_ production for uterine explants incubated with LPS (1 ug/ml), m-AEA (100 nM), URB597 (1 uM), LPS plus m-AEA (100 nM) or LPS plus URB597 (1 uM) for 24 h. ANOVA test. a, p<0.001 vs control; b, p<0.01 vs LPS; c, p<0.05 vs control; d, p<0.01 vs control. n = 7.

### Reverse Transcription Polymerase Chain Reaction (RT-PCR) Analysis

cDNA was synthesized from total RNA as described by Vercelli et al. [10]. Oligonucleotide primers for ***COX-1***, ***COX-2*** and ***β-actin*** were synthesized as described by Aisemberg et al. [1] and PCR cycle parameters were described in the same work. Oligonucleotide primers for ***CB1*** were 5′-ACCTGATGTTCTGGATCGGA-3′ (forward) and 5′-TGTTATCTAGAGGCTGCGCA-3′ (reverse) and for ***CB2*** were 5′-CCGGAAAAGAGGATGGCAATGAAT-3′ (forward) and 5′-CTGCTGAGCGCC CTGGAGAAC-3′ (reverse). Oligonucleotide primers for ***mPGES-1***, ***mPGES-2*** and ***cPGES*** were synthesized using Primer 3 Input free Software (v 0.4.0) [17]. For mPGES-1, 5′-GGGTCCCAGGAATGAGTACA-3′ (forward) and 5′-TTTCTGCTCTGCAGCACACT-3′ (reverse) were used; for mPGES-2, 5′-CAGGTACCAAGGCTGGATGT-3′ (forward) and 5′-CACCTGCAGGATGATGTACG-3′ (reverse) were used; and for cPGES, 5′-AAAATCCAGGCGATGACAAC-3′ (forward) and 5′-TTGGAAAGACTGGGAGGATG-3′ (reverse) were used. PCR products (COX-1, 449 bp; COX-2, 320 bp; β-actin, 392 bp; CB1,450 bp; CB2, 479 bp; mPGES-1, 237 bp; mPGES-2, 201 bp and cPGES, 194 bp) were separated on 1.5% agarose gel, stained with ethidium bromide, recorded under UV light with a digital camera Olympus C-5060 and analysed using the Image J software package (open source). Data were expressed as the relative amount of each PCR product versus β-actin mRNA.

### Quantitative Polymerase Chain Reaction (qPCR) Analysis

cDNA was synthesized from total RNA as described by Vercelli et al. [10]. Real-time quantitative PCR was performed with a Corbett-Rotor Gene system (Qiagen, Argentina) using EVA green (Biotium Inc., CA, USA) as the detection agent. Oligonucleotide primers for m-PGES-1 and β-actin were synthesized as described by Kubota et al. [18] and by Wang et al. [19], respectively. Specificity of the PCR reaction was controlled by the generation of melting curves. The relative gene expression levels were calculated using the comparative Ct (ΔΔCt) method [20]. Data was normalized to β-actin and mPGES-1 mRNA levels under control conditions (no m-AEA) were set to 1 (dotted line). Experiments were independently run three times. In each experiment, cDNA samples were performed in triplicate.

**Figure 3:**
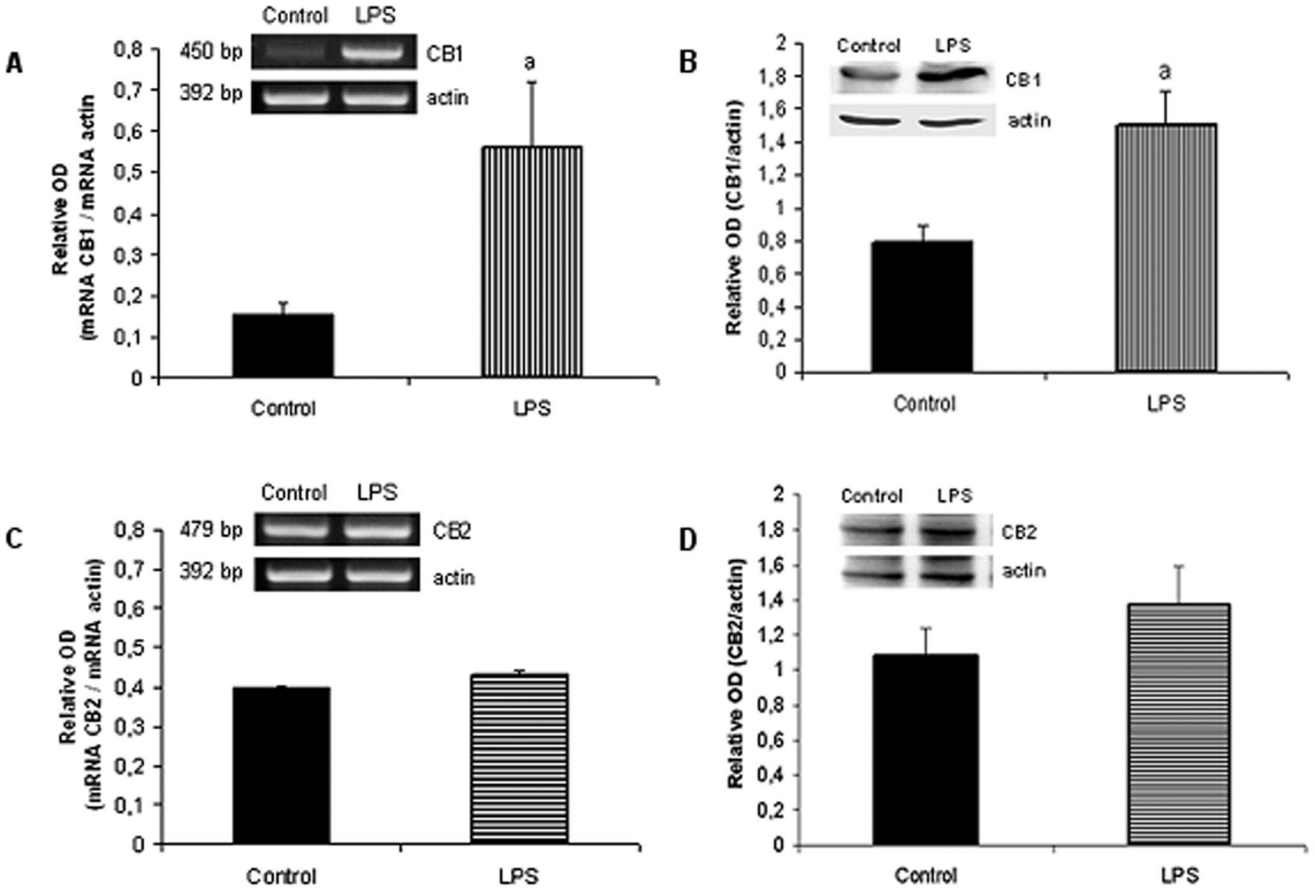
Effect of LPS on CB1 and CB2 levels. Densitometric analysis and expression of CB1 and CB2. Uterine explants were incubated without/with LPS (1 ug/ml) for 24 h and where subjected to RT-PCR and western blot analysis. Student t Test. a, p<0.05 vs control. Each gel was repeated three times with different samples. One representative gel/blot is shown.

### Western Blot Analysis

Tissues were homogenized and centrifuged as described by Vercelli et al. [21]. Samples were separated by electrophoresis and transferred to a nitrocellulose membrane. Blots were incubated overnight with the primary antibodies and 30 minutes with anti-β-actin, washed with buffer (10 mM Tris, 100 mM NaCl and 0.1% Tween 20, pH 7.5) followed by 1 h incubation with horse radish peroxidase-conjugated anti-rabbit secondary antibody, and developed using the enhanced chemiluminescence western blot system. Photographs of the membranes were taken using a digital camera and analysed using the Image J software package.

### PGE_2_ and PGF_2α_ Determination

The amount of PGE_2_ and PGF_2α_ were assayed in culture supernatants by specific radioimmunoassay (RIA) as reported by Farina et al. [22]. Briefly, specific antisera for both PGF_2α_ and PGE_2_ were used. Labelled [^3^H]-PGE_2_ and [^3^H]-PGF_2α_ were added to each tube. The incubation was performed for 90 min at 4°C. Bound and free radioligands were separated by dextran-coated charcoal and the tubes were centrifuged for 15 min at 2000×*g*. Results were expressed as pg PG/mg tissue (wet weight).

**Figure 4:**
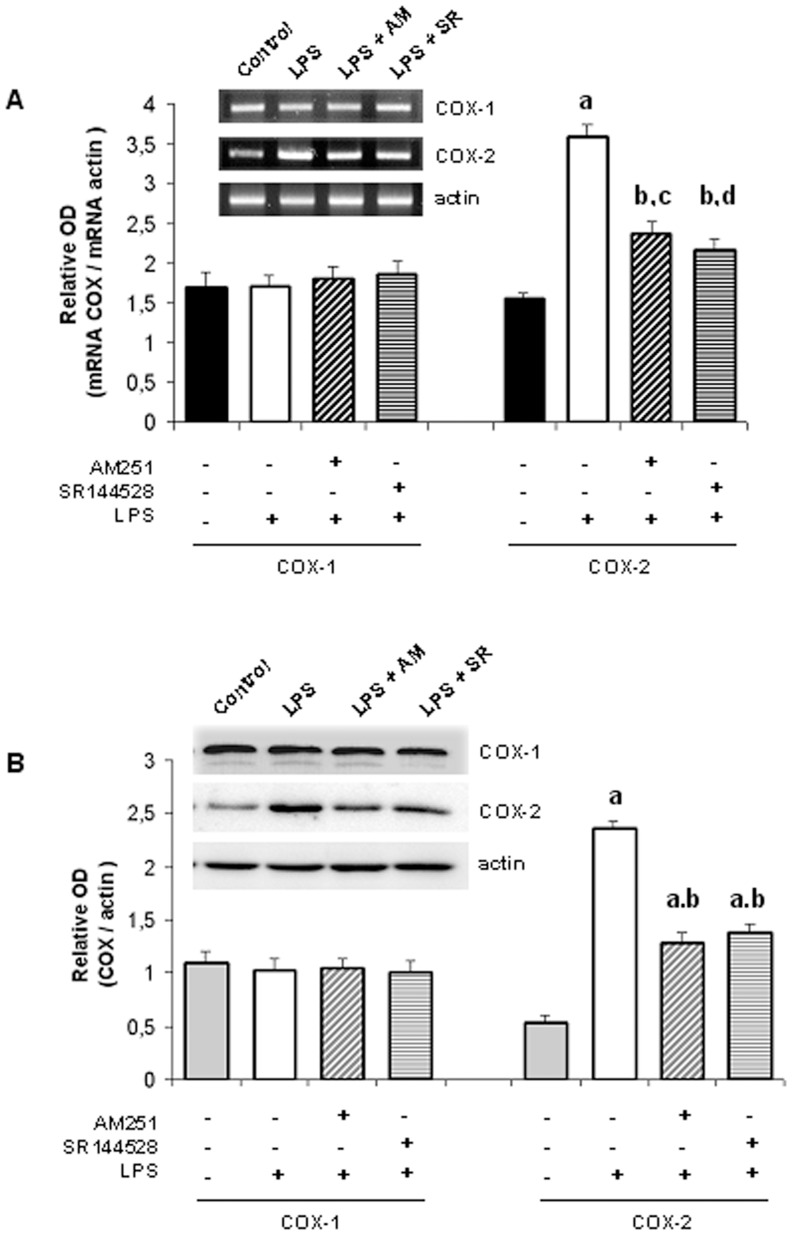
LPS-induced COX-2 expression is mediated by endocannabinoids. Densitometric analysis of A) mRNA and B) protein expression of COXs. Uterine explants were incubated with LPS alone (1 ug/ml), LPS plus AM251 (10 nM) or LPS plus SR144528 (10 nM) for 24 h. A) ANOVA test. a, p<0.001 vs control; b, p<0.001 vs LPS; c, p<0.01 vs control; d, p<0.05 vs control. n = 5. B) ANOVA test. a, p<0.001 vs control; b, p<0.001 vs LPS; n = 4. One representative gel/blot is shown.

**Figure 5:**
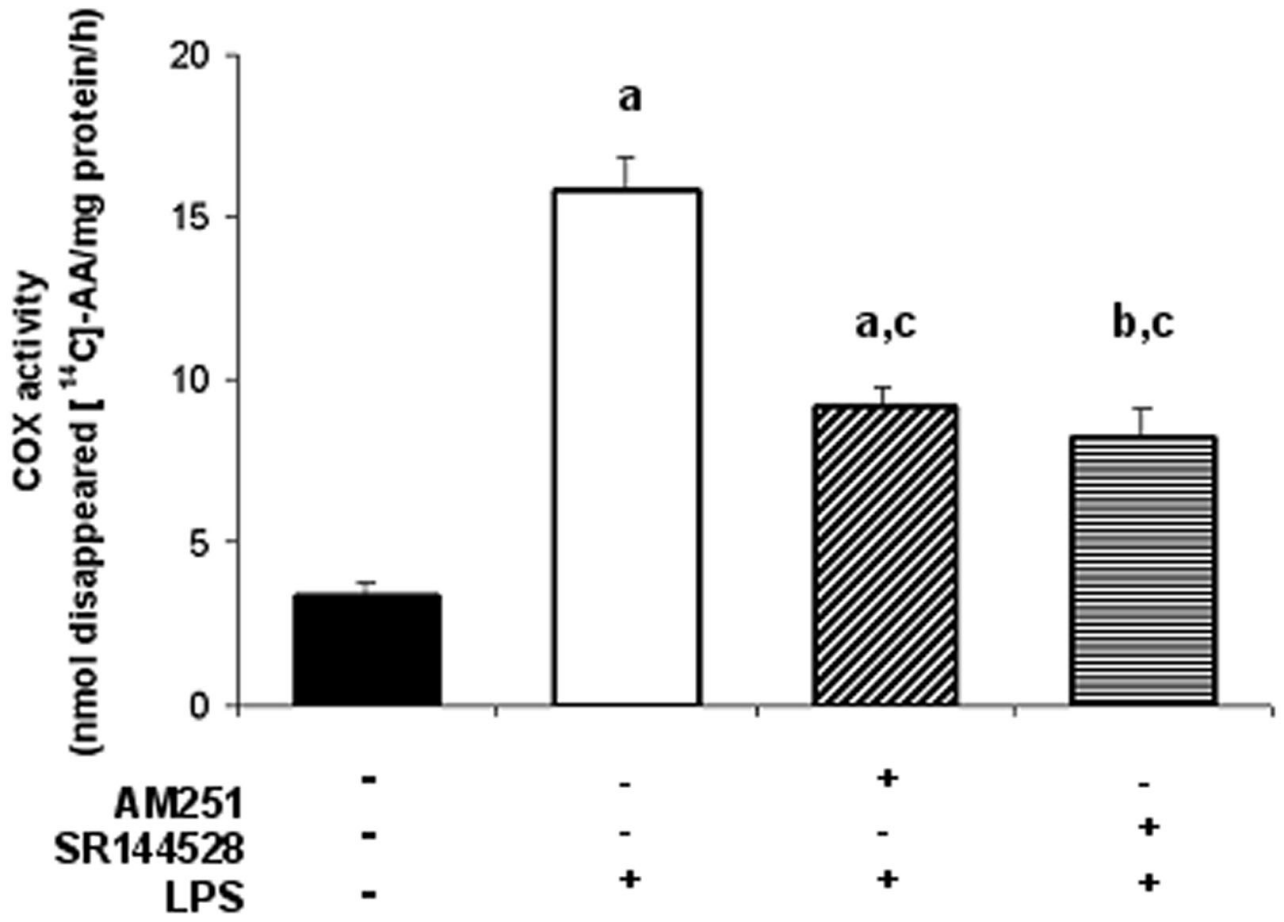
LPS-induced COX-2 activity is mediated by endocannabinoids. Uterine explants were incubated with LPS alone (1 ug/ml), LPS plus AM251 (10 nM) or LPS plus SR144528 (10 nM) for 24 h and COX-2 enzyme activity was determined by measuring the disappearance of the radiolabelled substrate [^14^C]-AA. Enzyme activity is reported as nmol of disappeared [^14^C]-AA/mg protein/h. ANOVA test. a, p<0.001 vs control; b, p<0.01 vs control; c, p<0.001 vs LPS; n = 4.

### Quantification of PGE_2_ metabolite (PGEM)

PGE_2_ metabolite was assayed in culture supernatants with the PGE metabolite EIA kit (catalog No. 14531; Cayman Chemical Co) according to the manufacturer’s recommendations. PGE_2_ is not chemically stable and is rapidly converted to its 13,14-dihydro-15-keto metabolite. The PGE metabolite assay kit converts all of the immediate PGE_2_ metabolites in the supernatant to a single stable derivative that could be easily quantified by EIA. PGEM levels are reported as pg PGEM/mg tissue (wet weight).

### Determination of PGE Synthase (PGES) Activity

PGES activity in uterine explants was measured by assessment of conversion of PGH_2_ to PGE_2_ as reported by Murakami et al. [23] with minor modifications. Briefly, tissues were homogenized in a 10 mM Tris-HCl buffer (pH = 8) and after centrifugation the supernatants were used as the enzyme source. One hundred micrograms of protein were incubated with 2 µg of PGH_2_ for 1 min at 24°C in 0.1 ml of 1 M Tris-HCl, pH = 8, containing 2 mM GSH. After terminating the reaction by the addition of 20 ul of HCl 6N, PGE_2_ content in the supernatants was quantified by radioimmunoassay. Results were expressed as pg PGE2/mg protein/h.

**Figure 6:**
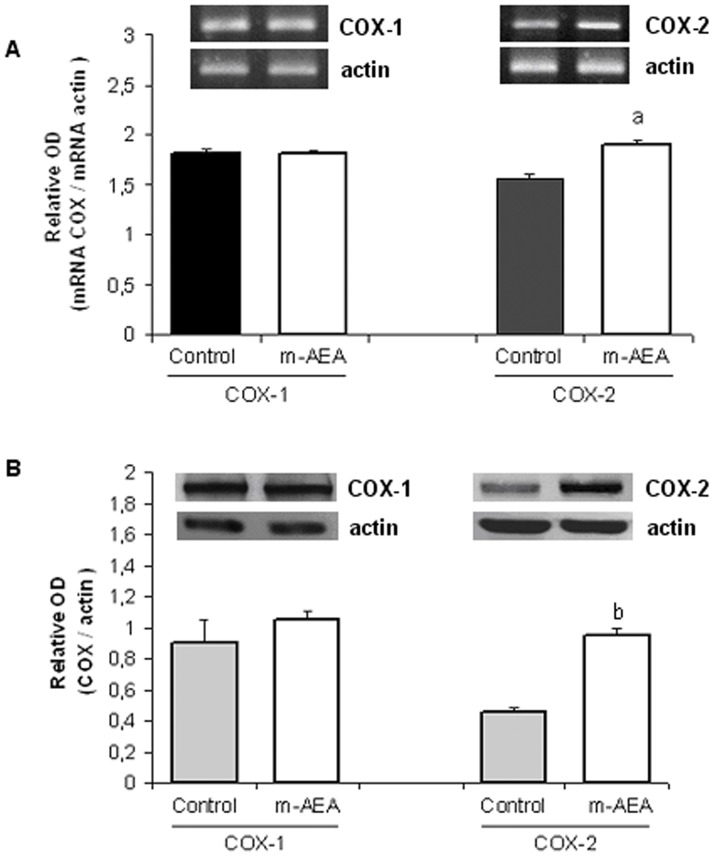
Effect of AEA on **COX-2 expression.** Densitometric analysis of A) mRNA and B) protein expression of COXs. Uterine explants were incubated without/with m-AEA (100 nM) for 24 h. Student t test. a, p<0.05 vs control; b, p<0.001 vs control. n = 4. One representative gel/blot is shown.

**Figure 7:**
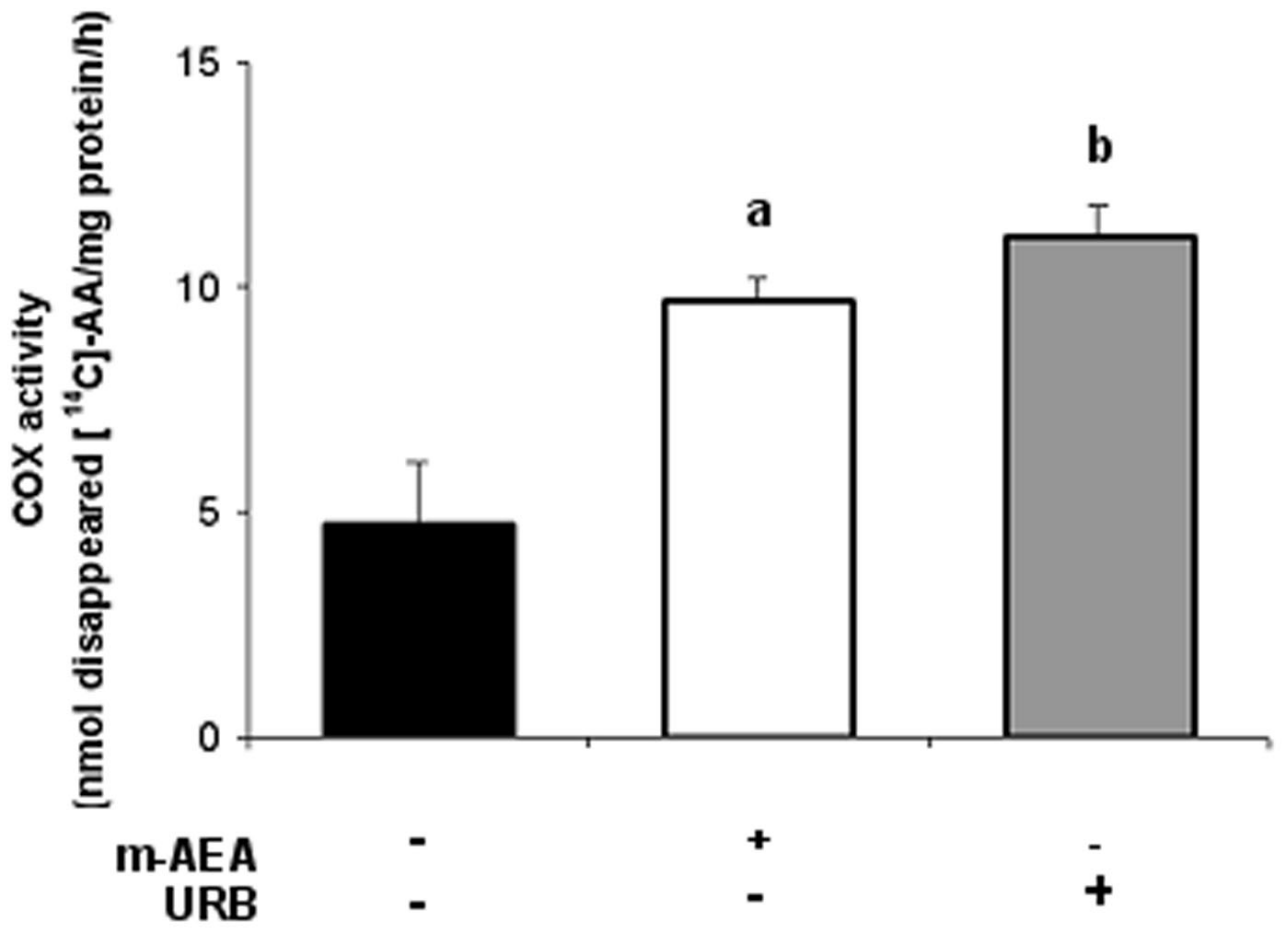
AEA increases COX-2 activity. Uterine explants were incubated with m-AEA (100 nM) or URB597 (1 uM) for 24 h and COX-2 enzyme activity was determined by measuring the disappearance of the radiolabelled substrate [^14^C]-AA. Enzyme activity is reported as nmol of disappeared [^14^C]-AA/mg protein/h. ANOVA test. a, p<0.05 vs control; b, p<0.01 vs control; n = 4.

### COX-2 Enzyme Activity Determination

COX-2 activity was determined by measuring the disappearance of the radiolabelled substrate [^14^C]-arachidonic acid using a modified method previously reported [24]. Briefly, 100 mg of uterine explants were homogenized in 1 ml cold fresh buffer (50 mM Tris-HCl and 1 mM EDTA, pH = 8), centrifuged at 20000 *x*g for 60 min at 4°C and supernatant was used for subsequent determination of enzyme activity. One hundred micrograms of protein were added to each incubation tube in cold fresh buffer to a final volume of 100 ul containing 110 000 d.p.m [^14^C]-arachidonic acid (AA, specific activity 53 mCi/mmol), either alone or with a selective COX-2 inhibitor (NS-398 1 µM). The sample and substrate mixture with a non-selective COX inhibitor (Indomethacin, [INDO] 1 µM) was used to determine the [^14^C]-AA disappearance values due to other enzymatic activities (lipoxygenase and/or epoxygenase) and non-enzymatic reactions [25]. The mixture was then incubated at 37°C for 30 min. Termination was achieved by addition of chloroform:methanol (1∶1, v/v). The unreacted [^14^C]-AA was resolved in the organic layer of a solvent system of ethyl acetate:hexane:acetic acid:distilled water (100∶50:20∶100 v/v) mixture. The distribution of radioactivity on the plate was counted in a scintillation counter by scraping off the corresponding spots detected in the plate. The area of each radioactive peak corresponding to AA was calculated and expressed as a percentage of the total radioactivity of the plates. For each sample, COX-1 activity was determined by calculating the rate of loss of [^14^C]-AA incubated with selective COX-2 inhibitor. Conversely, the COX-2 activity of each corresponding sample was determined by calculating the rate of loss of [^14^C]-AA incubated without selective COX-2 inhibitor, and subtracting from this value that of COX-1. The values of COX-2 and COX-1 were corrected by subtracting the [^14^C]-AA disappearance values due to other enzymatic activities and non-enzymatic reactions. Enzyme activity is reported as nmol of disappeared [^14^C]-AA/mg protein/h. The optimal reaction conditions were previously determined (data not shown).

**Figure 8:**
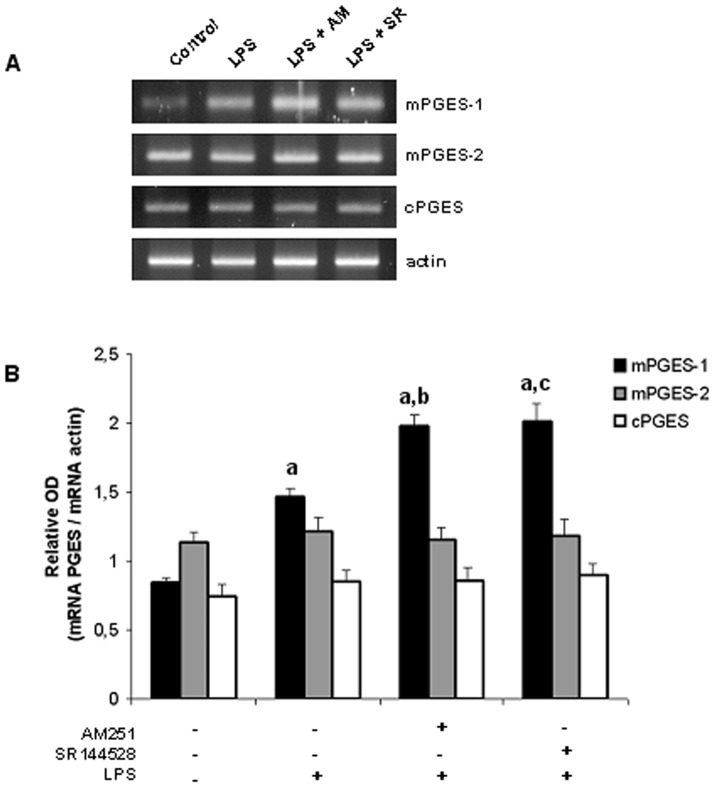
Endocannabinoids modulate mPGES-1 mRNA levels. Densitometric analysis and mRNA expression of PGES isoforms. Uterine explants were incubated with LPS alone (1 ug/ml), LPS plus AM251 (10 nM) or LPS plus SR144528 (10 nM) for 24 h. A) One representative gel is shown. B) Densitometric analysis. ANOVA test. a, p<0.001 vs control; b, p<0.01 vs LPS; c, p<0.001 vs LPS. n = 5.

**Figure 9:**
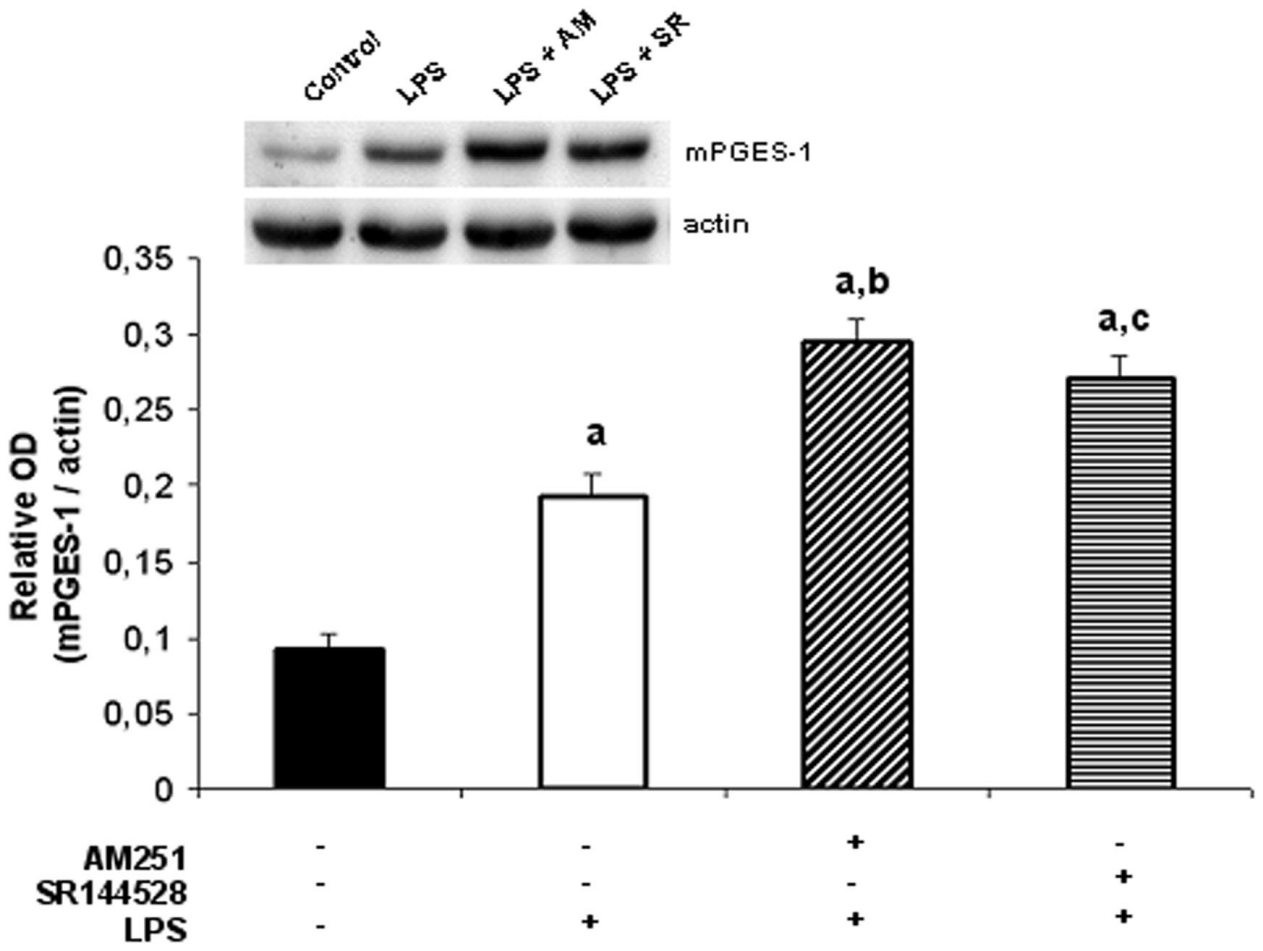
Endocannabinoids modulate mPGES-1 protein levels. Densitometric analysis and protein expression of mPGES-1. Uterine explants were incubated with LPS alone (1 ug/ml), LPS plus AM251 (10 nM) or LPS plus SR144528 (10 nM) for 24 h. ANOVA test. a, p<0.001 vs control; b, p<0.001 vs LPS; c, p<0.01 vs LPS. n = 4.

### Drugs, Chemical Reagents and Other Materials

LPS from *Escherichia coli* 05:B55, secondary horse radish peroxidase (HRP) conjugated antibody, anti-β-actin antibody, R-(+)-methanandamide, NADPH, and GSH were purchased from Sigma Chemical Co. (St Louis, MI, USA). AM251 was purchased from Tocris Cookson Inc. (Ellisville, MO, USA). SR144528 was kindly provided by Sanofi-Aventis. Indomethacin was obtained from Laboratorios Montpellier (Bs As, Argentina). TLC aluminum Silica Gel plates were purchased from Merck KGaA (Darmstadt, Germany). Western blotting reagents were obtained from Bio-Rad Laboratory. Anti-COX-1 and anti-COX-2 antibodies, PGH_2_, URB597 and Prostaglandin E Metabolite EIA kit were purchased from Cayman Chemical Co (Ann Arbor, MI, USA).The anti-mPGES-1 antibody was obtained from BD Biosciences (Franklin Lakes, NJ, USA). All other chemicals were analytical grade.

**Figure 10:**
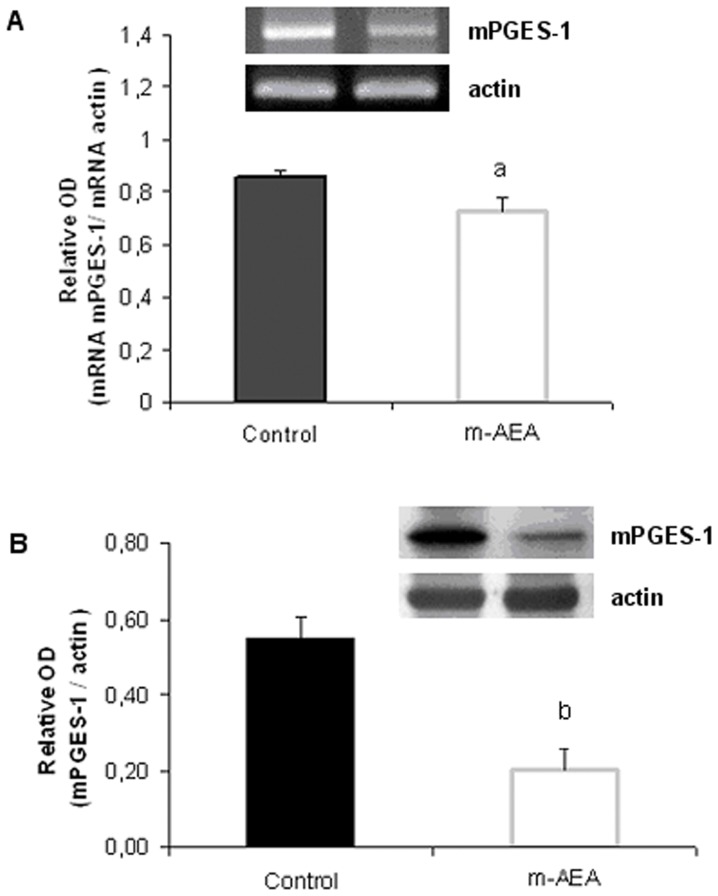
AEA decreases mPGES-1 mRNA and protein levels. Densitometric analysis of A) mRNA and B) protein expression of mPGES-1. Uterine explants were incubated without/with m-AEA (100 nM) for 24 h. Student t test. a, p<0.05 vs control; b, p<0.001 vs control. n = 5. One representative gel/blot is shown.

**Figure 11:**
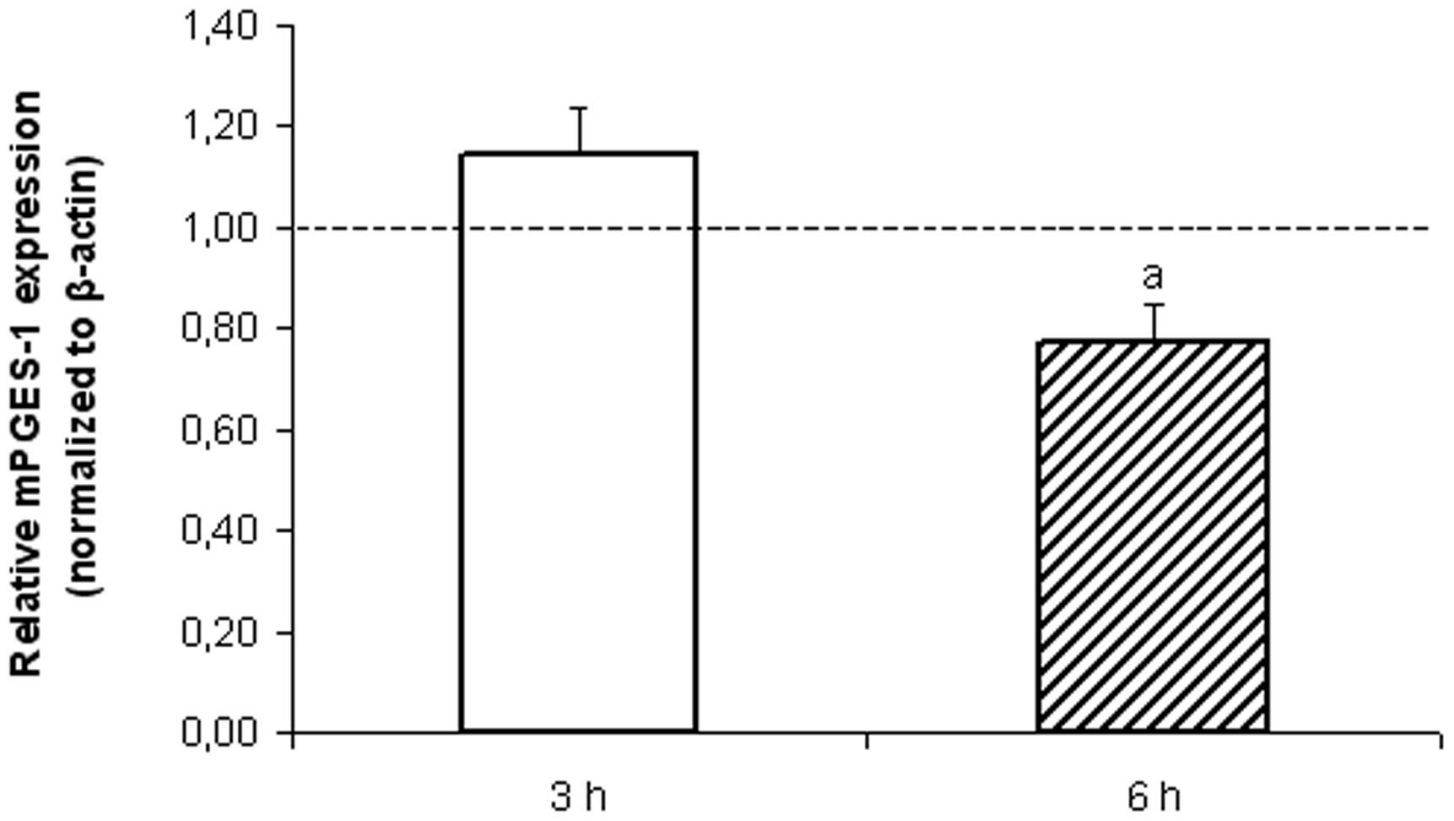
Quantitative real-time PCR analysis of mPGES-1 expression. Uterine explants were incubated without/with m-AEA (100 nM) for 3 h and 6 h and then subjected to qPCR analysis. Data was normalized against β-actin and mPGES-1 mRNA levels under control conditions (no m-AEA) were set to 1 (dotted line). Experiments were independently run three times. In each experiment, cDNA samples were performed in triplicate. Student t Test. a, p<0.05 vs control. n = 9.

### Data Analysis and Statistical Procedures

Statistical analysis was performed using the Graph Pad Prism Software (San Diego, CA, USA). Comparisons between values of different groups were performed using one-way ANOVA. Significance was determined using Tukey’s multiple comparison tests for unequal replicates or Student t Test. All values presented in this study represent means ± SEM. Differences between means were considered significant when p was 0.05 or less.

**Figure 12:**
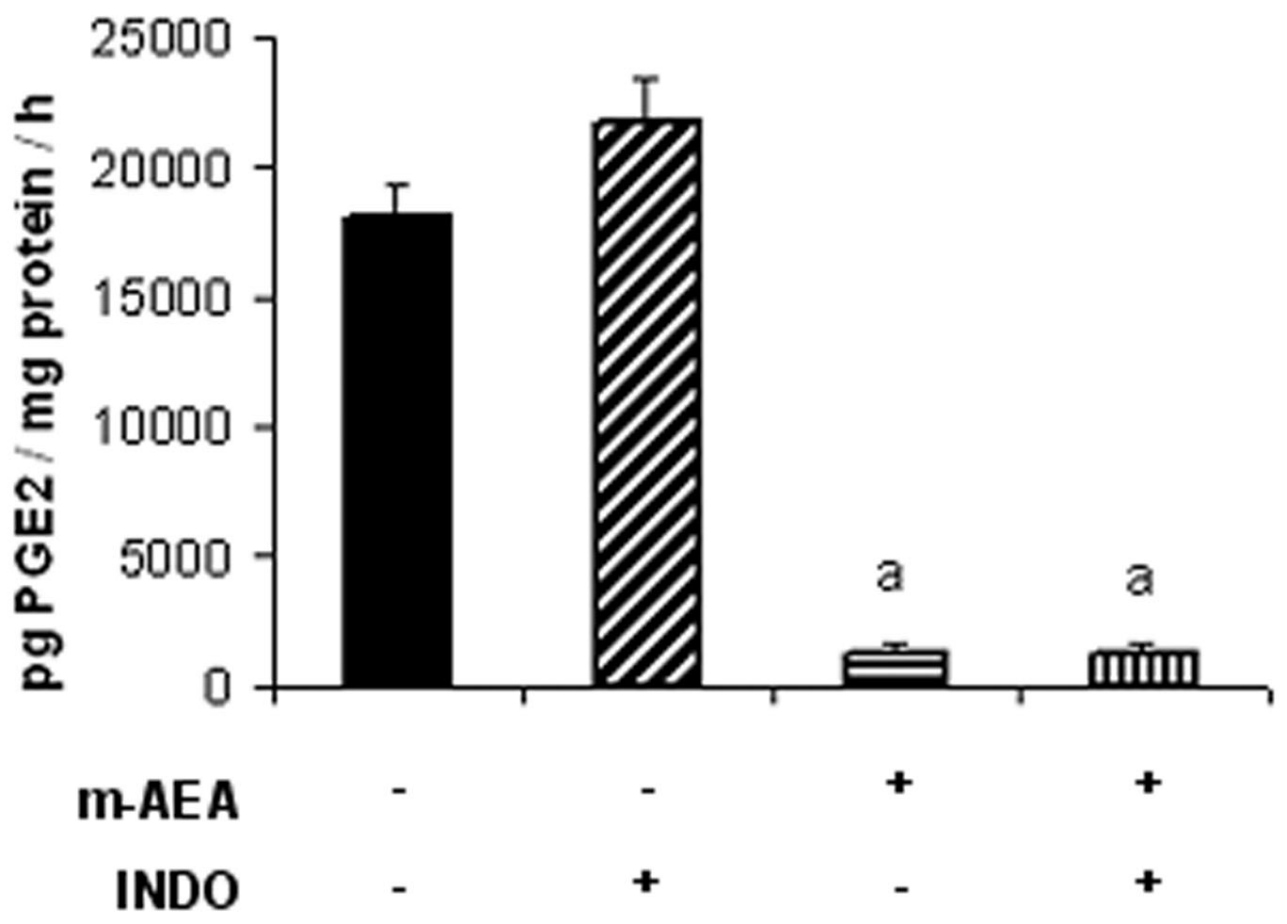
Effect of AEA on PGE synthase activity. Uterine explants were incubated with m-AEA (100 nM), INDO (1 µM) or m-AEA plus INDO for 24 h. Results were expressed as pg PGE2/mg protein/h. ANOVA Test. a, p<0.001 vs control or INDO. n = 4. PGE_2_ levels were assessed by RIA.

## Results

### LPS-induced PGE_2_ and PGF_2α_ Production is Mediated by Endocannabinoids

As mentioned before, previous results showed that *in vivo* administration of LPS increased PGE_2_ and PGF_2α_ production in the uterus of early pregnant mice [1]. Figure 1 (A and B) shows that uterine explants incubated for 24 h in the presence of LPS (1 µg/ml) induced PGE_2_ and PGF_2α_ production *in vitro*. To evaluate whether endogenous cannabinoids could modify LPS-induced PG production, uterine explants were incubated for 24 h in the presence of LPS (1 µg/ml), LPS plus AM251 (CB1 receptor antagonist) or LPS plus SR144528 (CB2 receptor antagonist) and PGE_2_ and PGF_2α_ levels were assessed by RIA. LPS-induced PGE_2_ synthesis was observed to be significantly increased when uterine explants were incubated with LPS and AM251 (10 nM) (Figure 1A). Nevertheless, incubation of the endotoxin with the CB2 receptor antagonist (10 nM) had no effect on PGE_2_ increased levels due to LPS in the same tissue (Figure 1A). Higher concentrations of the CB2 receptor antagonist were also used with the same results (data not shown). On the other hand, LPS-induced PGF_2α_ synthesis was partially but significantly decreased when uterine explants were incubated both with LPS and AM251 (10 nM) or with LPS and SR144528 (100 nM) (Figure 1B). PG levels remained unchanged when tissues were incubated either with AM251 or SR144528 alone. These findings suggest that endocannabinoids could exert opposite effects on PGE_2_ and PGF_2α_ biosynthesis, by inhibiting PGE_2_ production and increasing PGF_2α_ levels.

Among endocannabinoids AEA has been reported to regulate PG production in several tissues [12], [13]. Mitchell *et al.* have shown that AEA significantly reduced PGE_2_ production in human decidual tissue [11]; on the other hand, Someya *et al.* showed that AEA stimulates PGF_2α_ formation in PC12 cells [13]. Regarding LPS-induced PG production is mediated by endocannabinoids and considering that AEA could be involved in this process, we next analyzed if exogenous AEA could modify PG biosynthesis. First, uterine explants were incubated with methanandamide (m-AEA, a stable synthetic AEA analog) and PGE_2_ and PGF_2α_ levels were determined by RIA. We observed that explants incubated with m-AEA 100 nM showed a significantly increase of PGF_2α_ levels (Figure 2B), while PGE_2_ levels were significantly reduced (Figure 2A). We also used an inhibitor of AEA degradation (URB597, 1 uM) with the same results (Figure 2A and B). To test whether AEA mediates LPS-induced PG production, uterus was incubated in the presence of LPS (1 µg/ml) plus m-AEA (100 nM) or LPS (1 µg/ml) plus URB597 (1 uM) for 24 h and PG levels were assessed by RIA. Figure 2A shows that PGE_2_ production induced by LPS was partially but significantly decreased when tissues were incubated either with LPS plus m-AEA or with LPS plus URB597. On the other hand, PGF_2α_ production was significantly increased when tissues were incubated with LPS plus m-AEA (figure 2B).

**Figure 13:**
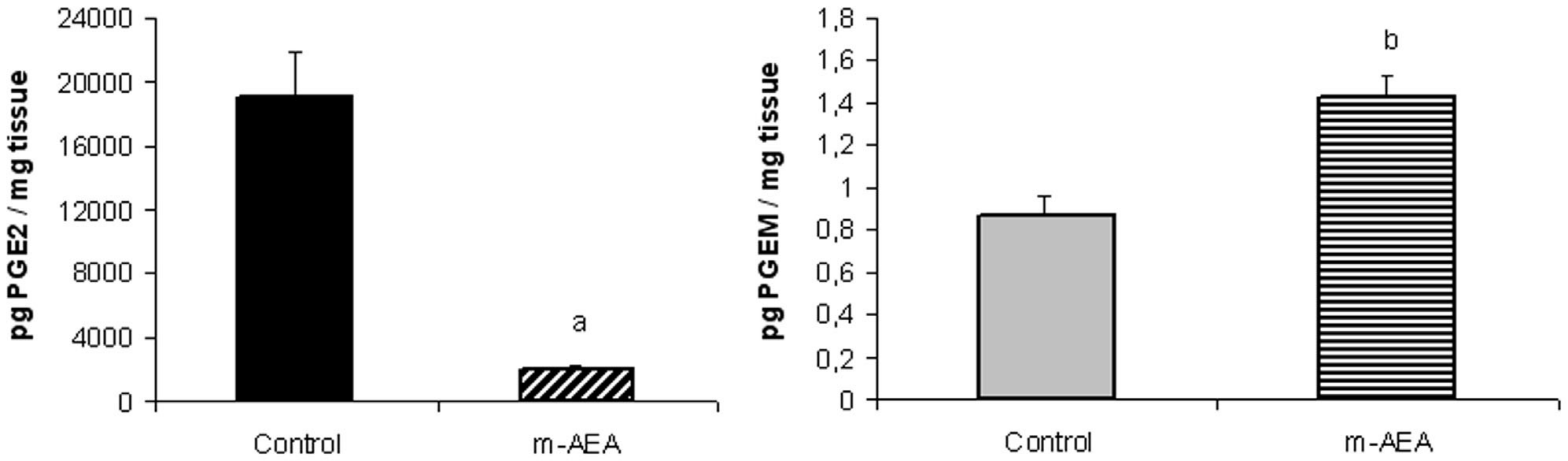
AEA augments PGE_2_ metabolite levels. Uterine explants were incubated without/with m-AEA (100 nM) for 24 h. A) PGE_2_ production assessed by RIA; B) PGEM quantification assessed by PGE metabolite EIA kit. Student t Test. a, p<0.001 vs control; b, p<0.01 vs control. n = 5. Both metabolites were assessed in the same sample. PGEM, Prostaglandin E_2_ metabolite.

### Effect of LPS on CB1 and CB2 mRNA and Protein Levels

The data showed in figure 1 suggest a possible involvement of CB1 and CB2 on PG production upon stimulation with LPS. In order to determine the effect of LPS on CB1 and CB2 expression in uterine explants, tissues were incubated for 24 h in the presence of LPS (1 µg/ml) and mRNA and protein levels were assessed. We observed the presence of both CB receptors, CB1 and CB2, in pregnant murine uterus. Furthermore, our results show that the endotoxin caused an increase in CB1 expression (Figure 3A and B) while CB2 levels remained unchanged (Figure 3C and D).

### LPS-induced PG Synthesis and Cyclooxygenase (COX) Expression is Mediated by Endocannabinoids

Since PG synthesis first involves the release of arachidonic acid which is converted to prostaglandin H_2_ (PGH_2_) by cyclooxygenase 1 (COX-1) or cyclooxygenase 2 (COX-2) enzymes, uterine explants were incubated for 24 h in the presence of LPS (1 ug/ml) alone, LPS plus AM251 (10 nM) or LPS plus SR144528 (100 nM) and COX-1 and COX-2 mRNA and protein levels were assessed. RT-PCR and western-blot analysis indicated that LPS significantly increased the expression of COX-2, whereas COX-1 mRNA and protein levels remained unchanged (Figures 4A and B). On the other hand, LPS-induced COX-2 expression (both mRNA and protein) was observed to be partially but significantly decreased when uterine explants were incubated either with LPS and AM251 or with LPS and SR144528 (Figure 4A and B). COX levels remained unchanged when tissues were incubated with AM251 or SR144528 alone (data not shown).

Regarding these results, we next evaluated COX-2 activity by measuring the disappearance of the radiolabelled substrate [^14^C]-arachidonic acid. Figure 5 shows a 3-fold increase in COX-2 activity when tissues were incubated with LPS alone. Moreover, LPS-induced COX-2 activity was partially but significantly decreased when uterine explants were incubated either with LPS and AM251 or with LPS and SR144528. These findings suggest that endocannabinoids mediate LPS-induced COX-2 activity.

As stated before, several lines of evidence indicate that both, cannabinoids and AEA, stimulate arachidonic acid (AA) release in several tissues [14], [15], [16]. Thus, we next analyzed if exogenous AEA could modify COX-1 and COX-2 mRNA and protein levels. RT-PCR and western-blot analysis indicated that m-AEA significantly increased the expression of COX-2, whereas COX-1 mRNA and protein levels remained unchanged (Figures 6A and B). We next evaluated COX-2 activity. Figure 7 shows a 2-fold increase in COX-2 activity when tissues were incubated either with m-AEA (100 nM) or URB597 (1 uM) alone. These results suggest that AEA could be mediating, at least partially, LPS-induced COX-2 activity.

### AEA: a Possible Role in PGE_2_ Metabolism

Our data show that AEA-induced PGF_2α_ production correlates with the increased expression and activity of COX-2. Nevertheless, decreased production of PGE_2_ induced by AEA is not explained by an augmentation of COX-2 expression and activity. In this sense, PGE_2_ levels could fluctuate due to inhibition of its synthesis, an increased catabolism or both. PGE_2_ synthesis involves the activity of PGE synthase (PGES) which catalyzes conversion of COX-derived PGH_2_ to PGE_2_. So far, three different isoforms have been identified with the capacity to synthesize PGE_2_ [26]. These include membrane-associated (or microsomal) PGE_2_ synthase-1 and –2 (mPGES-1 and mPGES-2, respectively), and cytosolic PGES (cPGES). Regarding this evidence, we first investigate whether endogenous cannabinoids could modify the expression of these enzymes and thus, we evaluated the effect of LPS plus CB receptor antagonists on uterine mRNA levels of PGES isoforms. Our results show that only mPGES-1 mRNA levels were significantly increased when uterine explants were incubated in the presence of LPS (Figure 8B). Moreover, LPS-induced mPGES-1 expression was observed to be significantly increased when uterine explants were incubated with LPS and AM251 (10 nM) or with LPS and the CB2 receptor antagonist (100 nM) (Figure 8B). We next evaluated if endocannabinoids could modulate mPGES-1 protein levels. Western blot analysis showed that LPS-induced mPGES-1 expression was significantly increased when tissues were incubated with LPS plus CB receptor antagonists (Figure 9).

As we mentioned before, AEA reduces PGE_2_ production in human decidual tissue [11]. Considering that LPS-induced mPGES-1 expression is mediated by endocannabinoids and regarding AEA could be involved in this process, we next analyzed if exogenous AEA could modify the expression of mPGES-1. Our results show that both mPGES-1 mRNA and protein levels were significantly reduced when uterine explants were incubated in the presence of m-AEA (100 nM) (Figure 10A and B, respectively).

Regarding the very small decline in mPGES-1 mRNA (although significant) and the substantial decrease in mPGES-1 protein we decided to make a more quantitative approach by performing a real time PCR at early times. We assessed mPGES-1 mRNA levels when uterine explants were incubated either for 3 h or 6 h in the presence of m-AEA (100 nM). We observed that mPGES-1 mRNA levels remained unchanged after 3 h incubation in the presence of m-AEA. Nevertheless, uterine explants incubated for 6 h in the presence of the synthetic analog reduced mPGES-1 mRNA levels up to nearly 25% (Figure 11).

We next investigate whether exogenous AEA could modify the production of PGE_2_ by modulating PGES activity. Thus, uterine explants were incubated in the presence of m-AEA (100 nM) alone or m-AEA plus indomethacin (INDO 1 µM [27], a non-selective COX inhibitor) and conversion of PGH_2_ to PGE_2_ was assessed by RIA. Incubation with INDO prevents COX-derived PGE_2_ *de novo* synthesis from AA release. We observed that m-AEA significantly decreased PGES activity (Figure 12). These findings suggest that AEA could be downregulating uterine PGE_2_ levels by inhibiting its synthesis.

Another enzyme which may influence the level and biological activity of PGs is 15-hydroxyprostaglandin dehydrogenase (15-PGDH). It is responsible for converting PGs into biologically inactive 15-keto derivative [28]. We first studied the effect of AEA on 15-PGDH mRNA and protein levels in uterine explants incubated with m-AEA 100 nM for 24 h. RT-PCR and western-blot analysis indicated that m-AEA did not modify 15-PGDH expression (data not shown). Next, we studied whether AEA could modify the production of PGE_2_ metabolite (PGEM, biologically inactive). In this sense, PGEM quantification was assessed in culture supernatants by Cayman’s PGE metabolite EIA kit as described in the *Materials and methods* section. At the same time, PGE_2_ levels were assessed in the same sample by RIA. Our results show that m-AEA (100 nM) significantly decreased PGE_2_ levels corroborating the results showed before, and at the same time, significantly increased PGEM production in the same samples (Figure 13).

In several tissues, including deciduas and fetal membranes, PGE_2_-9-ketoreductase (9-KPR) converts PGE_2_ into PGF_2α_ thus regulating PGE_2_/PGF_2α_ ratio in these tissues [29], [30]. Therefore, we studied the effect of AEA on 9-KPR activity in uterine explants incubated with m-AEA 100 nM for 24 h. We found that although we detected 9-KPR activity in the uterus of pregnant mice m-AEA did not modify it in this tissue (data not shown).

These findings suggest a possible role of AEA on PGE_2_ metabolism and, in this sense, AEA could be downregulating uterine PGE_2_ levels not only by inhibiting its synthesis but also by increasing its degradation.

## Discussion

Embryonic resorption induced by sepsis is viewed as a serious obstetric problem but it may also be seen from an evolutionary perspective as a pathophysiological mechanism to protect the maternal reproductive potential by expelling infected material. Prostaglandins are potent stimulators of myometrial contractility [31] and are used clinically to induce abortion and stimulate parturition [4]. Particularly, both PGE_2_ and PGF_2α_ stimulate myometrial contractility and are produced during labor by gestational tissues [32], [33]. Our previous results showed that *in vivo* administration of LPS increased PGE_2_ and PGF_2α_ production in the uterus of early pregnant mice. Also, we have observed that, in mice challenge with LPS, the *in vivo* administration of COX inhibitors prevented LPS-induced ER [1]. Collectively, these data suggest that although PGs are important paracrine regulators of uterine function in normal pregnancies, in an inflammatory setting such as sepsis, PGs have deleterious effects.

On the other hand, several lines of evidence show that LPS increases AEA levels [8], [9] and also that AEA modulates prostaglandin production in different tissues [11], [12], [13].

Based on the above evidence, we hypothesized that LPS-induced PG synthesis is mediated by endocannabinoids. Our results showed that endocannabinoids are involved in LPS-induced PG biosynthesis and also had different effects on PG biosynthetic pathways. While LPS-induced PGF_2α_ production was partially abrogated through both CB1 and CB2 receptors, LPS-induced PGE_2_ production was significantly increased when uterine explants were incubated with LPS plus a selective CB1 inhibitor (AM251) compared to explants incubated with LPS alone. On the other hand, CB2 receptor appeared not to be involved in LPS-induced PGE_2_ production. Considering that other groups’ findings show that, among endocannabinoids, AEA modulates PG biosynthetic pathways differently [11], [12], [13] we next evaluated whether AEA mediates LPS-induced PG production. Thus, we assessed uterine PGE_2_ and PGF_2α_ production in the presence of LPS plus a stable synthetic analog of AEA called m-AEA, or LPS plus an inhibitor of AEA degradation, URB597. Our findings showed that PGE_2_ production induced by LPS was partially but significantly decreased when tissues were incubated either with LPS plus m-AEA or with LPS plus URB597. On the other hand, PGF_2α_ production was significantly increased when tissues were incubated with LPS plus m-AEA. Together, these results suggest that AEA may be involved in LPS-induced PG production and that it modulates PG biosynthetic pathways differently for each PG. This is in accordance with the findings from other groups where it has been reported that AEA stimulates PGF_2α_ formation in PC12 cells [13] and also exerts a significant reduction in PGE_2_ production in human decidual tissue [11]. We have also shown that endocannabinoids mediate LPS-induced COX-2 activity, mRNA and protein expression and AEA could be involved in this process. Although we showed that mAEA had opposite effects on PGE_2_ and PGF_2a_ production, our previous results demonstrate that LPS induced the increase of PGs both *in vivo* [1] and *in vitro*. Also, LPS-induced COX-2 activity and expression was partially but significantly decreased when tissues were incubated with LPS and CB receptor antagonists. Taking this into consideration, it seems that AEA partially mediates LPS-induced PG production and therefore, we cannot rule out that other mechanisms (and/or other endocannabinoids) may be involved in this process.

On the other hand, we detected CB1 expression in the uterus of pregnant mice and also we reported for the first time the presence of CB2 in this tissue, contrary to other groups’ findings that suggest the absence of CB2 both in the oviduct and in the uterus of mice [34], [35]. Moreover, we have shown that LPS modulated CB1 mRNA and protein expression although CB2 levels remained unchanged. This is in accordance with the work of Matias et al. [36] and Do et al. [37] where they reported that LPS modulates CB1 and CB2 expression in dendritic cells.

Taking into consideration that AEA-induced PGF_2α_ production correlates with an increased expression and activity of COX-2 but decreased production of PGE_2_ induced by AEA is not in accordance with the regulation of COX-2 levels, we investigated whether endogenous cannabinoids could modify the expression of the three different isoforms with capacity to synthesize PGE_2_ identified so far, m-PGES-1, mPGES-2 and cPGES. Our results showed that only mPGES-1 expression was induced in the presence of LPS. This is in accordance with previous reports where a preferential coupling of COX-2 with mPGES-1 is shown in infectious response [38] and their expression is induced by pro-inflammatory mediators [23]. Moreover, LPS-induced mPGES-1 expression was observed to be significantly increased when uterine explants were incubated with LPS and CB receptor antagonists suggesting the participation of endocannabinoids in this process.

We also investigated the effect of exogenous AEA on different steps of PGE_2_ metabolic pathway. Our results showed that AEA downregulated PGE_2_ levels most likely due to inhibition of its synthesis and upregulation of its degradation. We have observed that AEA significantly reduced mPGES-1 mRNA and protein levels, which probably contribute to the decrease of PGE_2_ synthesis. These results are in accordance with Navarrete et al. [39] who reported that N-arachidonoyldopamine (NADA, another member of the endocannabinoid family) is a potent inhibitor of PGE_2_ production through a mechanism that involves reduction in the synthesis of mPGES-1 protein in LPS-activated microglia. Taking this evidence into consideration we cannot rule out that other endogenous CB1 and CB2 agonists (such as NADA and 2-AG, 2-arachidonoyl-glycerol) could also be involved in the action of LPS. The fact that AEA reduces PGE_2_ levels could be considered as an anti-inflammatory effect of this endocannabinoid.

On the other hand, although several groups have reported that uterine 15-PGDH activity is regulated by progesterone during the entire pregnancy [40], [41] this is the first time that an endocannabinoid modulation of PGE_2_ degradation is demonstrated.

The above reported data and our previous results [1] show that LPS induces PGE_2_ and PGF_2α_ production. Nevertheless, it is possible that PGE_2_/PGF_2α_ ratio more than the concentration reached by each of these PGs, may play a critical role in many biological functions, such as contractility. In fact, the altered ratio of PGE_2_/PGF_2α_ was described as one of the factors involved in the retention of fetal membranes in cattle [42], [43], [44]. In our case, where we studied PGE_2_ and PGF_2α_ participation in LPS-induced embryonic resorption [1] there always seemed to be a predominance of PGF_2α_ over PGE_2_. The difference in the amount of PGF_2α_ and PGE_2_ that reaches the uterus may play a role in the control of myometrial contractility. PGF_2α_ mediates myometrial contractions associated with the onset of labor [32]. Although it is less clearly established, PGE_2_ is thought to exert the opposite action and favor the establishment of pregnancy. Moreover, Slater et al. [45] have reported that PGE_2_ has anti-inflammatory and relaxatory effects on human myometrial smooth muscle.

Other groups have shown that LPS increases AEA production [8], [9] and in spite of the fact that we did not measure direct changes in endocannabinoid concentrations in culture media after LPS challenge, the use of CB specific antagonists and the use of m-AEA allow us to hypothesized that AEA could be involved in the mechanisms implicated in LPS-induced embryonic resorption. We propose that AEA participates in LPS-induced COX-2 expression and activity, increasing PGF_2α_ levels and downregulating PGE_2_ production modulating PG’s ratio in this tissue. AEA inhibitory effect on PGE_2_ production may be compensated by LPS stimulus (which may involve other endogenous CB receptor agonists and/or other mechanisms) and the final outcome is likely to be a PGE_2_/PGF_2α_ ratio that would favor myometrial contractions which in turn contribute to fetal expulsion in an inflammatory setting.

In conclusion, this study suggests that AEA acts as a pro-contractility agent modulating the production of PGs induced by LPS. This and our previous results showing that AEA participates in the increase of nitric oxide synthesis induced by LPS [10] demonstrate that this endocannabinoid could contribute to the mechanisms associated with pathological reproductive events such as septic abortion.

## References

[1] Aisemberg J, Vercelli CA, Billi S, Ribeiro ML, Ogando D (2007). Nitric oxide mediates prostaglandin deleterious effect on lipopolysaccharide-triggered murine fetal resorption.. Proc Natl Acad Sci USA.

[2] Michalek SM, Kiyono H, Babb JL, McGhee JR (1980). Inheritance of LPS nonresponsiveness and elevated splenic IgA immune responses in mice orally immunized with heterologous erythrocytes.. J Immunol.

[3] Cox SM, MacDonald PC, Casey ML (1988). Assay of bacterial endotoxin (lipopolysaccharide) in human amniotic fluid: potential usefulness in diagnosis and management of preterm labor.. Am J Obstet Gynecol.

[4] Hertelendy F, Zakar T (2004). Regulation of myometrial smooth muscle function.. Curr Pharm Des.

[5] De Petrocellis L, Cascio MG, Di Marzo V (2004). The endocannabinoid system: a general view and latest additions.. Br J Pharmacol.

[6] Wang H, Xie H, Sun X, Kingsley PJ, Marnett LJ (2007). Differential regulation of endocannabinoid synthesis and degradation in the uterus during embryo implantation.. Prost Other Lipid Med.

[7] Paria BC, Dey SK (2000). Ligand-receptor signaling with endocannabinoids in preimplantation embryo development and implantation.. Chem Phys Lipids.

[8] Maccarrone M, De Petrocellis L, Bari M, Fezza F, Salvati S (2001). Lipopolysaccharide downregulates fatty acid amide hydrolase expression and increases anandamide levels in human peripheral lymphocytes.. Arch Biochem Biophys.

[9] Liu J, Bátkai S, Pacher P, Harvey-White J, Wagner JA (2003). Lipopolysaccharide induces anandamide synthesis in macrophages via CD14/MAPK/phosphoinositide 3-kinase/NF-κB independently of platelet activating factor.. J Biol Chem.

[10] Vercelli CA, Aisemberg J, Billi S, Cervini M, Ribeiro ML (2009). Anandamide regulates lipopolysaccharide-induced nitric oxide synthesis and tissue damage in the murine uterus.. Reprod BioMed Online.

[11] Mitchell MD, Sato TA, Wang A, Keelan JA, Ponnanpalam AP (2008). Cannabinoids stimulate prostaglandin production by human gestational tissues through a tissue- and CB1-receptor-specific mechanism.. Am J Physiol Endocrinol Metab.

[12] Chen P, Hu S, Yao J, Moore SA, Spector AA (2005). Induction of cyclooxygenase-2 by anandamide in cerebral microvascular endothelium.. Microvascular Res.

[13] Someya A, Horie S, Murayama T (2002). Arachidonic acid release and prostaglandin F(2alpha) formation induced by anandamide and capsaicin in PC12 cells.. Eur J Pharmacol.

[14] Diaz S, Specter S, Vanderhoek JY, Coffey RD (1994). The effect of delta-9-tetrahydrocannabinol on arachidonic acid metabolism in human peripheral blood mononuclear cells.. J Pharmacol Exp Ther.

[15] Wartmann M, Campbell D, Subramanian A, Burstein SH, Davis RJ (1995). The MAP kinase signal transduction pathway is activated by the endogenous cannabinoid anandamide.. FEBS Lett.

[16] Hunter SA, Burstein SH (1997). Receptor mediation in cannabinoid stimulated arachidonic acid mobilization and anandamide synthesis.. Life Sci.

[17] Rozen S, Skaletsky HJ. Primer3 on the WWW for general users and for biologist programmers.. In: Krawetz S, Misener S (eds) Bioinformatics Methods and Protocols: Methods in Molecular Biology, Humana Press, Totowa, NJ. 2000. Pp 365–86..

[18] Kubota K, Kubota T, Kamei D, Murakami M, Kudo I (2005). Change in PGESs in microsomal PGES-1 knock out mice in a preterm delivery model.. J Endocrinol.

[19] Wang F, Wang J, Liu D, Su Y (2010). Normalizing genes for real-time polymerase chain reaction in epithelial and nonepithelial cells of mouse small intestine.. Anal Biochem.

[20] Livak KJ, Schmittgen TD (2001). Analysis of relative gene expression data using real-time quantitative PCR and the 2^−ΔΔCt^ method.. Methods.

[21] Vercelli CA, Aisemberg J, Billi S, Wolfson ML, Franchi AM (2009). Endocannabinoid system and nitric oxide are involved in the deleterious effect of lipopolysaccharide on murine decidua.. Placenta.

[22] Farina MG, Billi S, Sordelli MS, Ribeiro ML, Di Girolamo G (2006). Nitric oxide (NO) inhibits prostaglandin E2 9-ketoreductase (9-KPR) activity in human fetal membranes.. Prostaglandins Other Lipid Mediat.

[23] Murakami M, Naraba H, Tanioka T, Semmyo N, Nakatani Y (2000). Regulation of prostaglandin E2 biosynthesis by inducible membrane-associated prostaglandin E2 synthase that acts in concert with cyclooxygenase-2.. J Biol Chem.

[24] Zerani M, Dall’Aglio C, Maranesi M, Gobbetti A, Brecchia G (2007). Intraluteal regulation of prostaglandin F2α-induced prostaglandin biosynthesis in pseudopregnant rabbits.. Reproduction.

[25] Parthasarathy S, Wieland E, Steinberg D (1989). A role for endothelial cell lipoxygenase in the oxidative modification of low density lipoprotein.. Proc Natl Acad Sci USA.

[26] O’Banion MK (2010). Prostaglandin E_2_ synthases in neurologic homeostasis and disease.. Prostaglandins Other Lipid Mediat.

[27] Cella M, Leguizamón GF, Sordelli MS, Cervini M, Guadagnoli T (2008). Dual effect of anandamide on rat placenta nitric oxyde synthesis.. Placenta.

[28] Hansen HS (1976). 15-Hydroxyprostaglandin dehydrogenase. A review.. Prostaglandins.

[29] Schlegel W, Kruger S, Korte K (1984). Purification of prostaglandin E2–9-oxoreductase from human decidua vera.. FEBS Lett.

[30] Niesert S, Christopherson W, Korte K, Mitchell MD, MacDonald PC (1986). Prostaglandin E2 9-ketoreductase activity in human decidua vera tissue.. Am J Obstet Gynecol.

[31] Wiqvist N, Lindblom B, Wikland M, Wilhelmsson L (1983). Prostaglandins and uterine contractility.. Acta Obstet Gynecol Scand.

[32] Vane JR, Williams KI (1973). The contribution of prostaglandin production to contractions of the isolated uterus of the rat.. Br J Pharmacol.

[33] Gu W, Rice GE, Thorburn GD (1990). Prostaglandin E2 and F2 alpha in midpregnant rat uterus and at parturition.. Prostaglandins Leukot Essent Fatty Acids.

[34] Das SK, Paria BC, Chakraborty I, Dey SK (1995). The preimplantation mouse embryo is a target for cannabinoid ligand-receptor signaling.. Proc Natl Acad Sci USA.

[35] Wang H, Guo Y, Wang D, Kingsley PJ, Marnett LJ (2004). Aberrant cannabinoid signaling impairs oviductal transport of embryos.. Nat Med.

[36] Matias I, Pochard P, Orlando P, Salzet M, Pestel J (2002). Presence and regulation of the endocannabinoid system in human dendritic cells.. Eur J Biochem.

[37] Do Y, McKallip RJ, Nagarkatti M, Nagarkatti P (2004). Activation through cannabinoid receptors 1 and 2 on dendritic cells triggers NF-κB-dependent apoptosis: novel role for endogenous and exogenous cannabinoids in immunoregulation.. J Immunol.

[38] Jakobsson PJ, Thorén S, Morgenstern R, Samuelsson B (1999). Identification of human prostaglandin E synthase: a microsomal, glutathione-dependent, inducible enzyme, constituting a potential novel drug target.. Proc Natl Acad Sci USA.

[39] Navarrete CM, Fiebich BL, de Vinuesa AG, Hess S, de Oliveira AC (2009). Opposite effects of anandamide and N-arachidonoyl dopamine in the regulation of prostaglandin E and 8-iso-PGF formation in primary glial cells.. J Neurochem.

[40] Farina M, Ribeiro ML, Weissmann C, Estevez A, Billi S (2004). Biosynthesis and catabolism of prostaglandin F2 (alpha) are controlled by progesterone in the rat uterus during pregnancy.. J Steroid Biochem Mol Biol.

[41] Winchester SK, Imamura T, Gross GA, Muglia LM, Vogt SK (2002). Coordinate regulation of prostaglandin metabolism for induction of parturition in mice.. Endocrinology.

[42] Gross TS, Williams WF, Manspeaker JE, Lewis GS, Russek-Cohen E (1987). Bovine placental prostaglandin synthesis in vitro as it relates to placental separation.. Prostaglandins.

[43] Slama H, Vaillancourt D, Goff AK (1993). Metabolism of arachidonic acid by caruncular and allantochorionic tissues in cows with retained fetal membranes (RFM).. Prostaglandins.

[44] Horta AE, Chassagne M, Brochart M (1986). Prostaglandin F_2α_ and prostacyclin imbalance in cows with placental retention: new findings.. Ann Rech Vet.

[45] Slater DM, Astle S, Woodcock N, Chivers JE, de Wit NC (2006). Anti-inflammatory and relaxatory effects of prostaglandin E2 in myometrial smooth muscle.. Mol Hum Reprod.

